# Capillary-Driven Water Transport by Contrast Wettability-Based Durable Surfaces

**DOI:** 10.1021/acsami.3c03840

**Published:** 2023-05-26

**Authors:** Theodoros Dimitriadis, Luca Stendardo, Irene Tagliaro, Anna Maria Coclite, Carlo Antonini, Tanmoy Maitra

**Affiliations:** † Institute of Solid-State Physics, Graz University of Technology, Graz 8010, Austria; ‡ Department of Materials Science, University of Milano-Bicocca, Via R. Cozzi 55, 20125 Milano, Italy; § Department of Engineering, FT Technologies (UK) Ltd., Sunbury-on-Thames TW16 7DX, United Kingdom

**Keywords:** laser surface texturing, wettability contrast, polymer coating, surface durability, water transportation

## Abstract

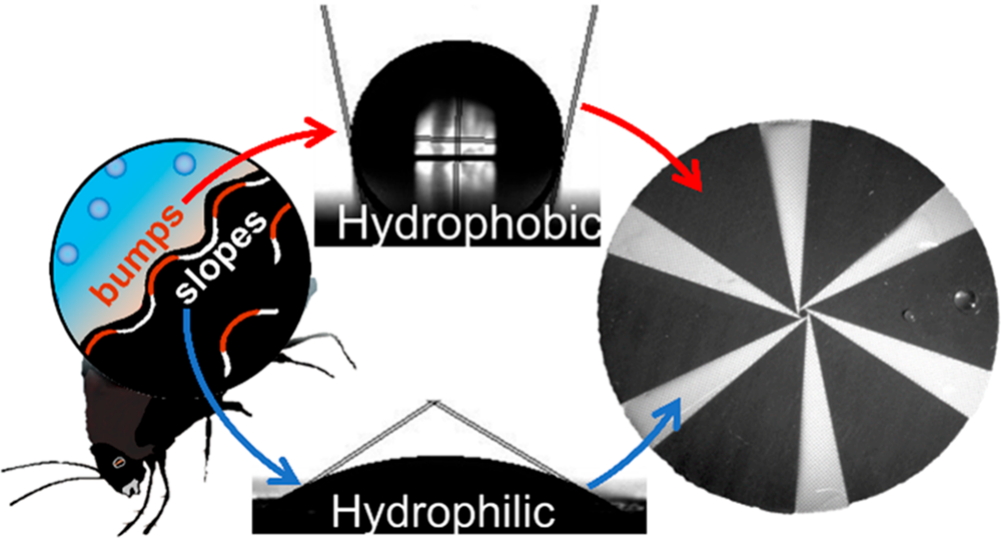

Controlling water transport and management is crucial for continuous and reliable system operation in harsh weather conditions. Passive strategies based on nonwetting surfaces are desirable, but so far, the implementation of superhydrophobic coatings into real-world applications has been limited by durability issues and, in some cases, lack of compliance with environmental regulations. Inspired by surface patterning observed on living organisms, in this study we have developed durable surfaces based on contrast wettability for capillary-driven water transport and management. The surface fabrication process combines a hydrophobic coating with hard-anodized aluminum patterning, using a scalable femtosecond laser microtexturing technique. The concept targets heavy-duty engineering applications; particularly in aggressive weather conditions where corrosion is prevalent and typically the anodic aluminum oxide-based coating is used to protect the surface from corrosion, the concept has been validated on anodic aluminum oxide coated aluminum alloy substrates. Such substrates with contrast wettable characteristics show long-term durability in both natural and lab-based artificial UV and corrosion tests where superhydrophobic coatings tend to degrade.

## Introduction

Surfaces with contrast wettability, alternating hydrophobic and hydrophilic areas, are widespread in nature as they are effective in water transport and management.^1−3^ As an example, *Stenocara* sp., a tenebrionid beetle, possesses a combined texture and pattern of wettability on its exoskeleton, consisting of nonwaxy tips and waxy slopes, to harvest water from the coastal fog-laden wind in Namib’s arid desert. The beetle basks in the fog, standing upside down on top of sand dunes: water first collects in small droplets, which then roll down the beetle’s back toward its mouth, under gravity.^1^ Inspired by this and other examples, it is possible to create contrast wettability surfaces for many engineering applications where the directional transport of liquids is required.^4−8^ To achieve such contrast with wettable surfaces, various techniques based on wet chemistry and dry etching have been proposed,^9−13^ followed by surface modifications.^14−16^ Notwithstanding the plethora of techniques available, such approaches did not lead to commercial products due to mainly two reasons: first, durability issues in real-world applications^17−21^ and, second, production upscaling concerns related to the use of chemicals or processes that are not environmentally friendly (according to REACH, RoHS list, and other environmental regulations).^22,23^ To overcome these shortcomings, in the present study, we present a biomimetic design based on contrast wettability to control water transport on aluminum alloy, one of the most common engineering substrates used in industry; this can be applied in applications where guided water transport is essential, such as proton exchange membrane fuel cells, digital microfluidics, thermal management of electronics, and water harvesting applications.^4−8,24,25^ The novel surface structure, fabricated by applying a durable powder coating of a fluorine-based polymer to anodized aluminum followed by a microtexturing technique by a scalable femtosecond laser system,^23,24,26−28^ leverages open capillary-driven water transport: the combination of microscale surface wettability laser textures with millimeter-precise radial tracks enables a self-propelled capillary-driven water transport mechanism. Such an approach allows the fabrication of durable and environmentally friendly surfaces, as validated in both laboratory-based artificial tests and one-year-long natural weathering in Miami, Florida, USA, a location with 4 times more aggressive weathering conditions than in Europe,^29^ which is a crucial factor for extensive evaluation studies before using it on production scale.

## Results and Discussion

### Water Transport on Contrast Wettability Surface

**Figure 1:**
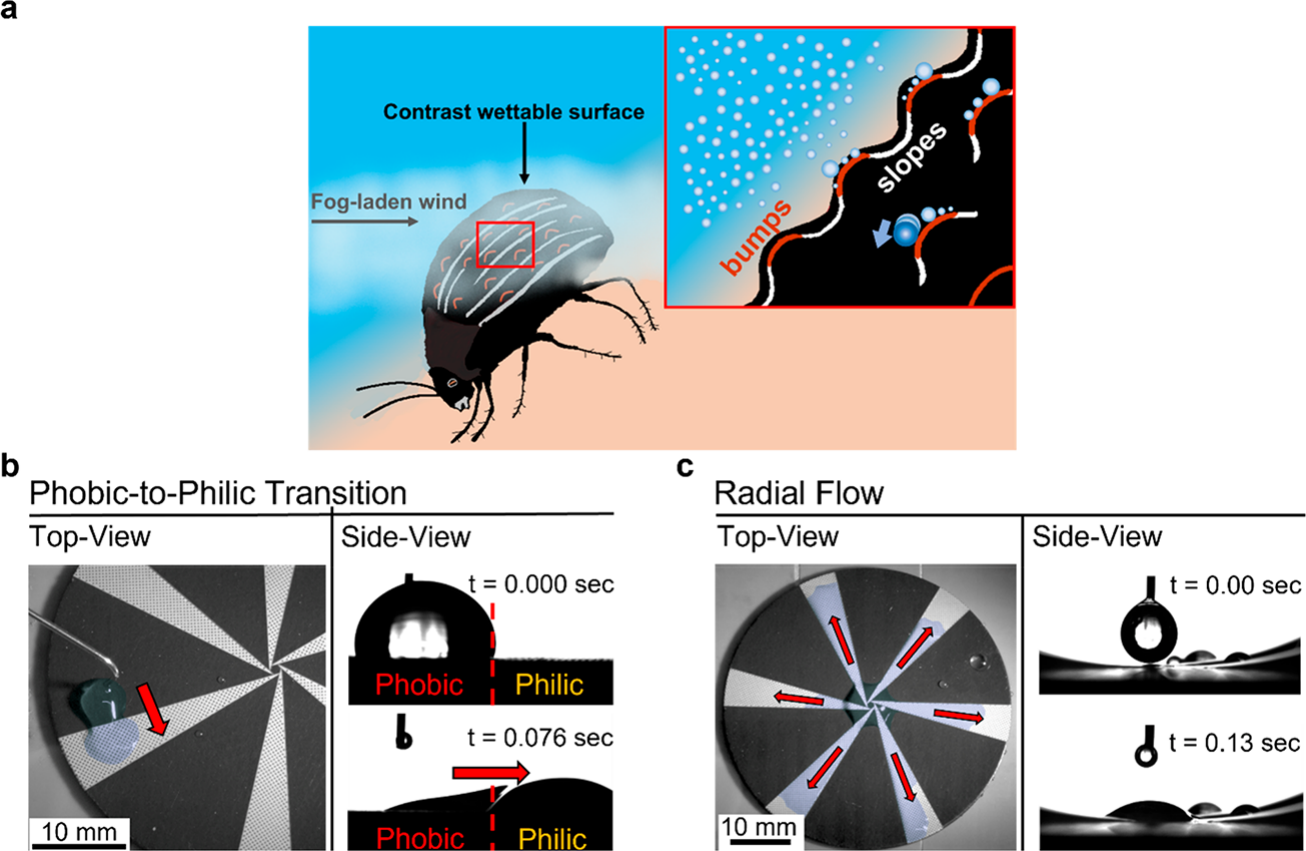
Working principles of the bioinspired surface on an application scale. (a) Namib desert beetle collects droplets of water using its contrast wettability patterned back, which allows it to guide water to its mouth. Its back consists of hydrophilic (nonwax) bumps set among hydrophobic (waxy) areas (inset). Its back design and natural forces (wind and gravity) help the beetle to harvest water. As the droplets coalesce, they eventually become large enough to roll off the hydrophilic island. At that moment the capillary force that attaches the droplet to the surface is overcome by gravity. As a result, the droplet rolls to the hydrophobic area, eventually leading to its mouth. (b, c) Top and side views of the radial flow and phobic-to-philic transition, respectively, two key mechanisms that enable efficient displacement of accumulated water. A single tapered track can continuously remove up to 10 mL/min in completely wet conditions.

The innovative concept of contrast wettability is exploited by the Namib desert beetle to adeptly collect water from coastal fog-laden wind and direct it to its mouth as it stands upside down (Figure 1a). Similarly, the textured surface we have fabricated is made of alternating hydrophobic (perfluoroalkoxy) areas next to sections of hard-anodized aluminum alloy with laser-fabricated microtextures, which are conversely hydrophilic and allows better control of the surface roughness. The combination of hydrophilic and hydrophobic areas results in contrast wettability surfaces (Figure 1b,c).

As in the case of the beetle, the biomimetic design presented here is based on two working principles to direct water on a solid surface: the phobic-to-philic transition and the outward radial flow through hydrophilic tracks. Figure 1b illustrates the first key mechanism for enhanced water collection and removal, which involves drops on the hydrophobic surface transitioning into the wet, hydrophilic region (phobic-to-philic transition). Subsequently, due to the radial flow, water is passively pumped outward on the hydrophilic tracks. By growing droplets close to the hydrophobic/hydrophilic junction (flow direction perpendicular to the junction line), volumes up to 50 μL could be transported by a single hydrophilic track, leaving the hydrophobic surface practically free from water. The complete measurements of the absorption of individual droplets on the hydrophilic tracks and further details can be found in the section S1 of Supporting Information.

The flat-contrast wettability surface creates a radial flow (Figure 1c) on the laser-microtextured hydrophilic tracks, allowing for water transport from the center to the outer region of the horizontal sample surface through the use of surface tension and Laplace pressure differences (discussed in detail in the section “Effect of Channel Geometry”). The outer edge of the contrast wettability surface remains uncoated, leaving the naturally hydrophilic hard-anodized aluminum exposed. Once the aqueous liquid reaches the outer edge, the water is drawn away from the hydrophilic tracks due to this lack of coating. The hydrophilic surface on the edge of the contrast wettability surface acts, therefore, as a gravity-assisted sink to remove the excess water from the hydrophilic tracks. The performance of the contrast wettability surface was tested by establishing a continuous water flow on the wet hydrophilic tracks. Tests demonstrated that up to 10 mL/min can flow in a single hydrophilic track. Considering that the full surface includes six tracks, the total water displacement capacity is measured to be 60 mL/min, based on dropwise water application. However, it is important to note that this capacity should not be interpreted as the maximum capacity of the surface to handle continuous exposure to large volumes of water. Nonetheless, the measured capacity exceeds the water accumulation rate of heavy-rainfall conditions in a natural environment,^30^ which suggests that the surface may be effective in managing water in engineering applications.

### Surface Characterization

**Figure 2:**
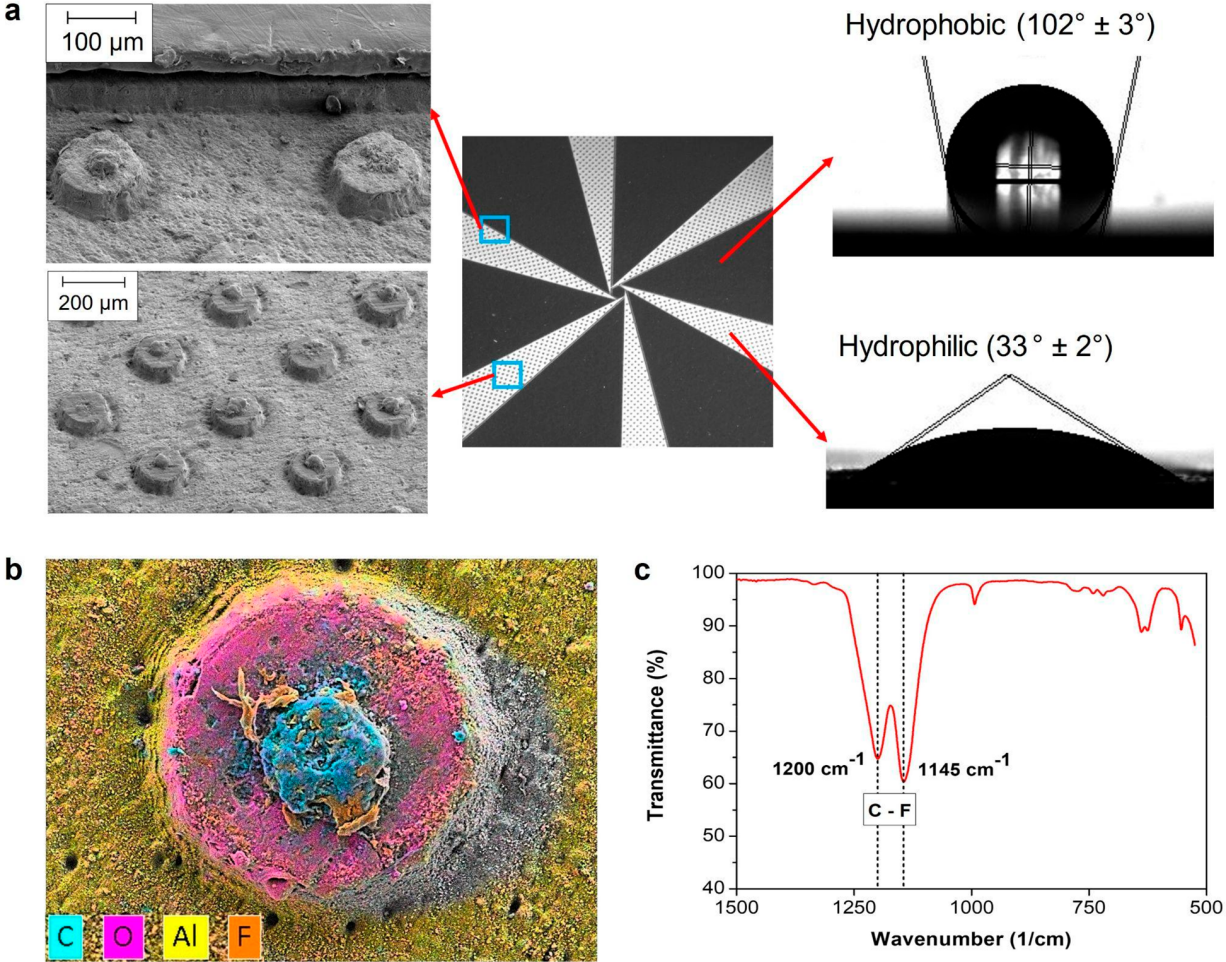
Characterization of the contrast wettability surface. (a, middle) Contrast wettability surface comprising the laser-ablation, tapered tracks of hard anodized aluminum, bounded by the polymer islands both on the sides of the tracks and in the center of the sample. (Top-left) SEM images of the micropillar structure next to the hydrophobic/hydrophilic junction and (bottom-left) the micropillar structure on the hydrophilic surface away from the junction. (Top- and bottom-right) Water contact angles measured on both hydrophobic and hydrophilic surfaces, respectively. (b) EDX mapping of the elemental composition on the laser-ablated micropillar structures. The colors refer to the dominant peak by EDX. Each element is separately presented in Figure S3. (c) FTIR analysis of the polymer coating. The absorption band in the region between 1100 and 1250 cm^–1^ corresponds to vibration modes of CF_*x*_ (*x* = 1–3) groups.^31^

To facilitate the investigation of the contrast wettability surface properties, surfaces have been prepared on circular aluminum alloy substrates with a hard-anodized aluminum oxide coating. Figure 2a shows a top-down image of the contrast wettability surface, including SEM images and contact angle θ measurements. The base material is coated with a hydrophobic, fluorine-based polymer [Teflon perfluoroalkoxy (PFA)]. After the coating process, the laser-ablation technique was used to generate the hydrophilic/hydrophobic pattern by selectively removing the surface polymer and microtexturing the exposed substrate.

The hydrophilic tracks do not meet at a single point in the center of the sample (see Figure 2a, center). Instead, they intersect each other in a manner so that they define a hydrophobic center (as shown in Figure S2). The six tapered tracks diverge over the sample by an angle (Ψ), which is set to 15°. The effect of this angle on the surface tension-driven force is explained in the section “Effect of Channel Geometry”. During the laser-ablation process, a cylindrical-shaped microstructure was applied to the tracks (see Figure 2a, SEM images) to increase the total surface area, reduce the contact angle thus increasing hydrophilicity,^32^ and simultaneously decrease the pinning effects of large volumes of water.^33^ The micropillars have an average height of 68 ± 2 μm, the pitch (center to center) distance is 346 ± 2 μm, and the pillar top surface diameter is 143 ± 2 μm. The average ablation depth (from the polymeric surface to the pillar bottom) is 141 ± 2 μm. The polymer-coating shows an average contact angle of θ = 102° ± 3°, while for the microstructured aluminum tracks the θ was found to depend on the humidity conditions. In wet conditions (which are representative of heavy rainfall and in high humidity environments, see methods section for details) the measured contact angle on hydrophilic tracks is θ = 33° ± 2° (refer to section S3 of the Supporting Information for the variation of the apparent θ in different conditions). The behavior of changing wetting properties depending on the humidity conditions can be explained by taking a closer look at the composition of the pillar tops: from the energy-dispersive X-ray analysis (EDX, Figure 2b) it is evident how the micropillar tops show a high concentration of carbon and fluorine. Due to the laser ablation procedure (refer to section S6 of the Supporting Information), it has been noticed that pyrolyzed polymer residue (see Figure 2c for the FTIR analysis of the polymer coating) is formed on top of the pillars.^34^ The presence of combustion residues reduces the hydrophilic characteristic of the tracks in dry environmental conditions. Nonetheless, during heavy rain and high humidity conditions, the impinging water droplets and condensation of water vapor displace the air pockets in the microstructure, which enhances hydrophilicity. Under these conditions, the tracks exhibit a strong hydrophilic character, irrespective of the polymer residues on the pillar top, which proves useful in creating a continuous flow of water from the center of the contrast wettability surface toward the outer edge.^34^ The capillary-driven flow on hydrophilic tracks results in exceptional water removal.

### Effect of Channel Geometry

The droplet movement along the tapered track is driven by the net capillary force and the Laplace pressure imbalance. The magnitude of the capillary force is determined by the difference between the contact angles on the polymer coating (θ_p_) and that on the hydrophilic sections (θ_h_). The higher the difference between θ_p_ and θ_h_, the higher is the capillary force.

**Figure 3:**
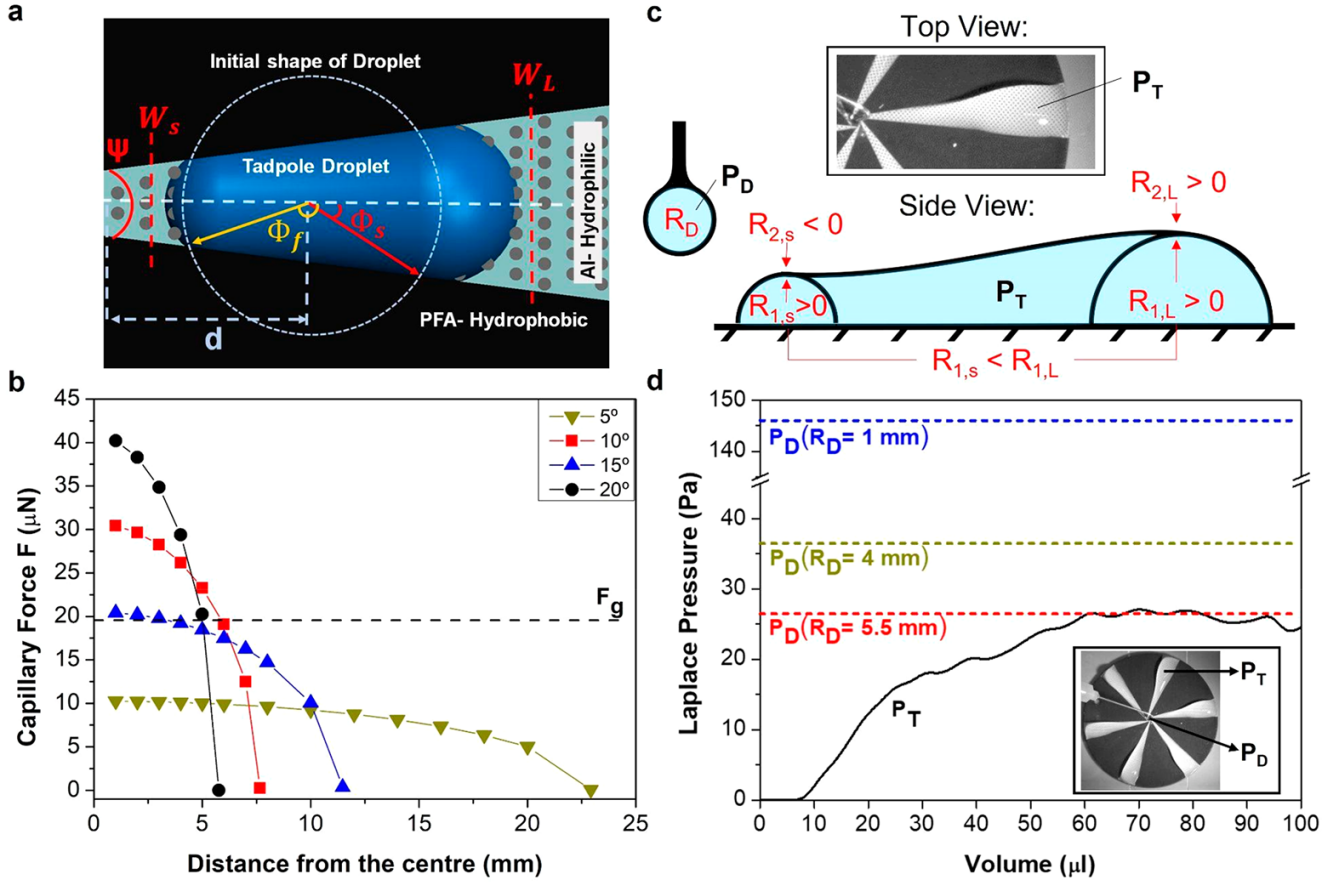
Physics of transporting a droplet along a tapered track. (a) Schematic of the initial circular droplet placed over hydrophobic/hydrophilic junctions. The angles of the contact line at the junctions (Φ_f_ and Φ_s_) are relevant for the capillary force. Due to that, the initial spherical droplet will eventually move to the hydrophilic tapered track and the covered distance depends on the angle Ψ and takes the form of a tadpole droplet. (b) The graph shows the link between the net capillary force, *F*, and the head angle of the wedge, Ψ. The wider (large Ψ) the track, the higher the capillary force is achieved close to the center of the sample. In order to achieve a maximum capillary force along the tapered track, it is recommended to design a track with variable wedge angles. *F*_g_ is the gravitational force. (c) Schematic of the radii of curvature for water accumulation on hydrophilic tapered tracks, which provides insight into the Laplace pressure-based water transport mechanism. The diverging shape of the hydrophilic tapered tracks constrains the radius of curvature *R*_1_, resulting in a water transfer toward the larger portion of the track. The inset figure shows a top view of the bulge of water developing over the hydrophilic track, keeping a liquid footprint from the center to the edge of the contrast wettability surface. The shape of the bulge was analyzed to explain the Laplace pressure difference-driven water displacement. (d) Laplace pressure *P*_T_ on a single tapered track as a function of accumulated volume. The dotted lines represent Laplace pressures of spherical droplets of different radii.

While moving on the hydrophilic tapered track (Figure 3a), the droplet takes an elongated shape, following the edges of the track geometry. However, to determine the initial driving force caused by surface tension when a droplet first touches the hydrophilic tapered track, the contact line can be assumed as circular. As shown in Figure 3a, the center of the droplet is at a distance *d* from the center of the sample (i.e., the narrow end of the hydrophilic track). Before taking an elongated shape, the droplet contact line touches the hydrophobic/hydrophilic boundary at two points: The first point makes an angle Φ_f_ with the centerline of the hydrophilic track, and the second point makes an angle Φ_s_. Assuming that the contact angle changes abruptly at the hydrophobic (θ_p_)/hydrophilic (θ_h_) junction, the driving force (*F*) for a water droplet of radius *R* can be estimated by the following equation:^35^
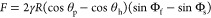
1The larger the divergence angle Ψ of the track, the larger is the term (sin Φ_f_ – sin Φ_s_); therefore, a higher driving force is achieved. Assuming a constant diameter of the droplet, a greater divergence angle Ψ implies higher initial driving forces due to surface tension near the center of the sample (Figure 3b). However, the driving force rapidly decreases as the droplet moves toward the edge of the hydrophilic track as shown in Figure 3b. On the other hand, by narrowing (small Ψ) the hydrophilic track, the driving force is lowered, and therefore the water droplet moves slowly toward the edge of the surface.^35^ In this work, the hydrophilic tapered channels have a constant diverging angle (Ψ) of 15°, which allows the capillary force to guide the droplet toward the half of the hydrophilic track.

The capillary force, *F*, describes the radial pull that single droplets experience at the first contact with the hydrophilic track. In a scenario where the precipitation rate is increased, more and more water accumulates on the hydrophilic tracks. In this case, a continuous water bulge is formed on the hydrophilic track, from the center to the edge of the contrast wettability surface (Figure 3c, top inset). It is observed in these conditions that whenever a new droplet coalesces with the water bulge on the hydrophilic sections, a waterfront propagates on the hydrophilic track. The hydrophilic track remains completely flooded but continues to displace water from the center toward the outer region of the sample. This phenomenon can no longer be explained with the capillary force previously presented eq 1, but in this case, a Laplace pressure differential will drive the excess water toward the outer edge of the contrast wettability.

To investigate the Laplace pressure-driven radial flow in more detail, a test sample with the contrasting wettability pattern is first immersed in water to ensure complete wetting and then droplets are deposited at the center of the substrate, where the hydrophilic tracks converge. During the deposition of the water drops, the sample was placed on a horizontal surface. The water flows on the hydrophilic tracks and forms a bulge over the hydrophilic tracks. The evolution of the water shape is recorded by a camera (refer to section S4 of the Supporting Information), allowing us to measure of the surface curvature of the bulge and calculate the Laplace pressure within the water accumulations.

In this experiment, millimetric drops (drop diameter of 2 mm, *V* ≈ 5 μL) are continuously injected at the center of the sample at a flow rate of 100 μL/min with the aid of a syringe pump and a syringe. Before the drop touches the surface, the Laplace pressure inside the droplet (*P*_D_) is ∼146 Pa (Figure 3d, blue dotted line). When the deployed droplet coalesces with water on the hydrophilic tracks, this pressure (*P*_D_*)* is exerted on the previously accumulated water, initiating a liquid flow. The liquid driving force is determined by the Laplace pressure difference between the droplet (*P*_D_) and the water accumulation on the hydrophilic tracks (*P*_T_). During dropwise water deployment, there is no continuous water flow from the needle to the hydrophilic tracks, and for this reason, the volume of water on the tracks between two successive droplet deployments is considered stationary. The Laplace pressure value inside the water accumulation is therefore equal at every point, making it possible to calculate *P*_T_ at the most convenient location.

*P*_D_ was calculated by measuring the droplet radius, while *P*_T_ was calculated by computing the two radii of curvature *R*_1,L_ and *R*_2,L_ of the water shape (Figure 3c):
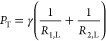
2The pressure curve *P*_T_ (Figure 3d) is plotted as a function of the collected water volume and is compared to the Laplace pressure lines of spherical droplets of various radii. The pressure differential exists up to a droplet radius of about 5.5 mm, which matches the size of large rain droplets found in nature during rainfall.^36^ It is therefore expected that releasing any droplet with a radius below 5.5 mm at the center of the contrast wettability surface will cause a driving force that can displace the excess liquid from the point of deployment onto the hydrophilic sections.

As explained earlier in this section, the Laplace pressure term *P*_T_ can be calculated at any point of the water volume by considering the two characteristic radii of curvature. The sum of the reciprocals of the radii of curvature can therefore be considered constant over the entire hydrophilic track.
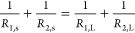
3Figure 3c shows a schematic representation of the shape of the water retention. It can be noted how the water line is convex close to the center of the contrast wettability surface, near the point of water deployment, and the curvature radius *R*_2,s_ can therefore be considered negative. On the other hand, further toward the outer regions of the hydrophilic track, *R*_1,L_ and *R*_2,L_ assume positive values (the water line is concave).

For *P*_T_ to be equal in the whole water volume, the following relation holds:
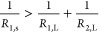
4In order to respect the constant *P*_T_ condition, the tapered shape of the hydrophilic tracks transfers water toward the outer region of the contrast wettability surface.

In conclusion, the inherently hydrophilic hard-anodized aluminum substrate, in combination with the microtextured surface, contrast wettability, and geometry of the tracks, enhances the capillary driving force and Laplace pressure imbalance and promotes the advancement of a water droplet along the hydrophilic/hydrophobic boundary toward the edge of the substrate.

### Artificial and Natural Weathering Tests

**Figure 4:**
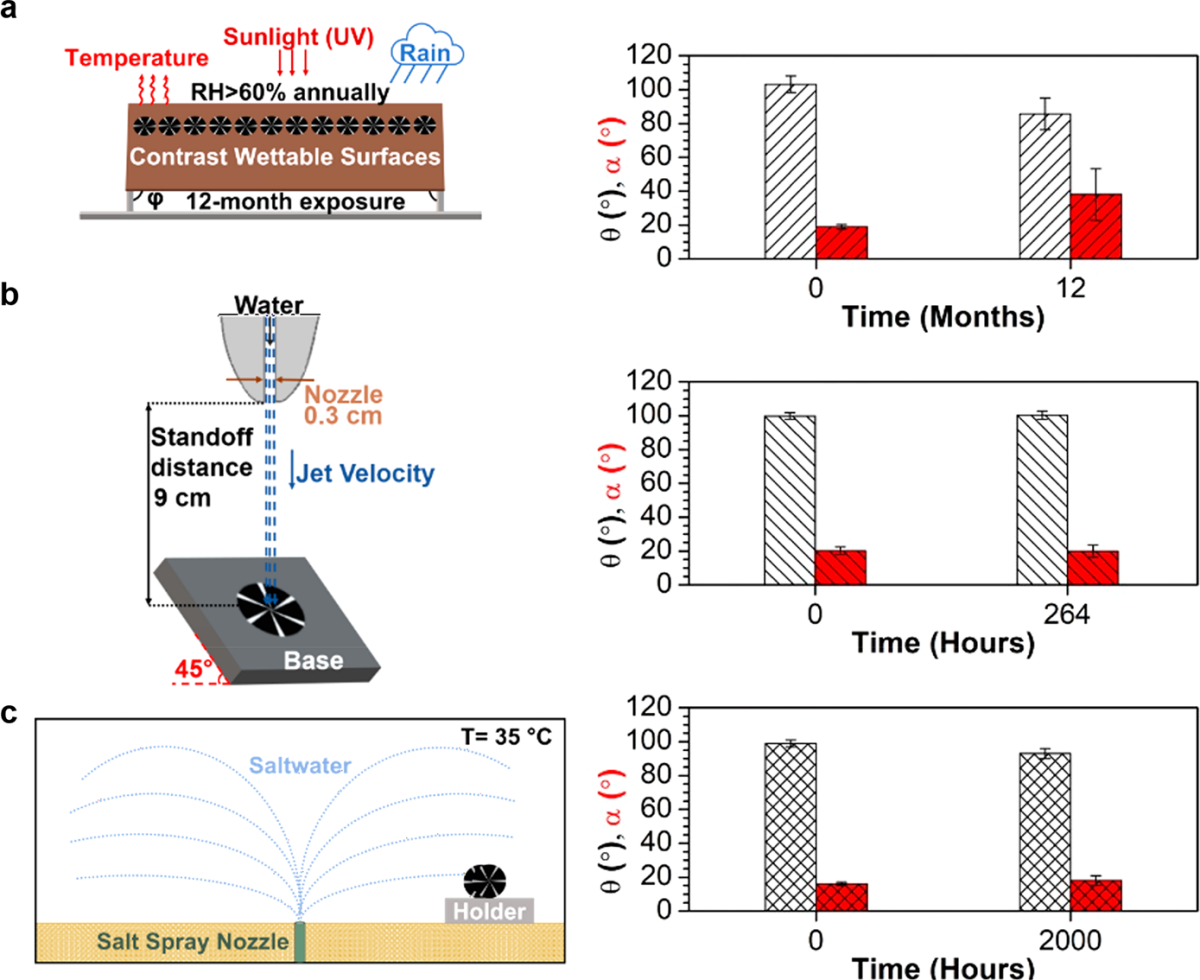
Validation of the wetting properties of contrast wettability surface through natural weathering and artificial tests. (Left-side) The figure shows the natural weathering and artificial tests, (right side) as well as the water contact angle and slide angle measurements, respectively, for each test on the coatings before and after exposure. (a) 12 contrast wettability tokens have been exposed in Miami, Florida, USA, environmental conditions (see meteorological data in Figure S6) for up to 12 months. The graph presents the measured values of the contrast wettability surface samples before and after 12 months of exposure. Especially for the contrast wettability surfaces, which contain polymeric material, the most important factor of degradation is the sunlight. Therefore, the stage is tilted by an angle φ with respect to the month in order to receive the highest amount of solar radiation (see section “Methods and Apparatus”). (b) A contrasting wettable sample was exposed to the water jet, and its wetting properties were measured at the beginning and end of the test. (c) Contrast wettability surfaces exposed in the corrosion chamber. The data presented here are done on the hydrophobic polymeric islands, and they are the average value of 10 measurements. The corrosion chamber operated at 35 ± 2 °C.

To demonstrate the durability of the contrast wettability surface in natural weathering conditions, we chose one of the world’s most aggressive benchmark locations, Miami, Florida, U.S., since in this region materials are subject to high sunlight radiation, high temperatures, high annual rainfall, high humidity (relative humidity more than 60% annually; see Figure S6), and salty wind (Figure 4a). The degradation of the coating and laser microtextures was studied at the end of each month over a period of 12 consecutive months.

After 12 months of continuous outdoor exposure, the water contact angle on the hydrophobic coated part was decreased to θ ∼ 85° from the initial value of θ ∼ 103°. However, despite this degradation, the surface maintained its ability to water transportation.

Additionally, the durability of the contrast wettability surface is evaluated against a high-speed waterjet test (Figure 4b). The samples were mounted on an inclined (φ = 45°) plate, which was exposed to a waterjet with impinging velocity *V*_0_ of approximately 12 m s^–1^ (We ∼ 6100 and Re ∼ 36 200) for up to 264 h. In Figure 4b, the average contact angle, θ, and slide angle, α, are presented before and after the completion of the test. The reported degradation is ∼1° for both angles. Accordingly, the values measured every 24 h, 10 measurements for each sample (contrast wettability), in terms of θ and α are presented in Figure S9. ASTM B117 test has been performed to laser microtextured contrast wettability surface. In this experiment, the test sample has been exposed directly to the corrosion environment for up to 2000 h while the temperature inside the chamber is kept constant at 35 ± 2 °C (Figure 4c). The contrast wettability surface showed a slight degradation from θ ∼ 99° to θ ∼ 93° and α was increased ∼2°. Regarding the contrast wettability surface, all of the sample’s alternative surfaces (hydrophilic and hydrophobic) maintained their properties. No mechanical degradation was observed in microscale features.

## Conclusions

In this study, we designed and developed a durable and reproducible bioinspired contrast wettability surface,^37^ machined by a femtosecond industrial laser. In addition, we textured circular micropillar arrays to make the fabrication process easier and controllable compared to random textures and microtextures of any other shapes, thus making this suitable for large volume production and enhancing wettability contrast with the hydrophobic coated parts leading to faster and efficient pumpless water transportation. In wet conditions such as high humidity or heavy rainfall, results showed that the contrast wettability surface is able to displace up to 60 mL/min in the radial direction, exceeding the rainfall rate of what is considered “heavy-rainfall” conditions.^30^ The surface modifications effectively displace water by utilizing the capillary forces (due to surface tension and Laplace pressure), which are controlled by the contrast wettability and the diverging shape of the hydrophilic tapered tracks. We have also demonstrated the improved durability of the contrast wettability surface through exposure to harsh weather conditions, both natural and artificial.

After 12 months of exposing the samples to harsh weathering conditions, the contrast wettability surface showed a minor degradation in terms of its wettability properties, which nonetheless did not affect the transport efficiency.

As such, the surface wettability contrast can be of interest for a variety of practical applications, where water management represents a critical issue, spanning from atmospheric water harvesting and collection to water elimination from engineering devices and sensors operating in rain conditions. As an outlook, we envision the development of such strategies even in icing conditions at low temperatures, where a surface approach may help to mitigate ice formation and growth.

## Experimental Section

### Materials and Coatings Fabrication

#### Substrates

Aluminum (Al) alloy with hard anodized aluminum oxide coating (∼40–50 μm) has been used as the substrate.

#### Coating and Microtexturing

A hydrophobic Teflon-like coating that offers θ = 100° and α = 15° has been used as a hydrophobic coating. An industrial femtosecond laser has been used to create microscale features on the anodized aluminum substrates.

### Methods and Apparatus

#### Approach for Surface Morphology Characterization

The wettability of the surfaces was characterized via static contact angle (θ) and slide angle (α) measurements using the sessile drop method with an in-house instrument. Water droplets up to 50 μL volume were deposited gently and vertically in atmospheric conditions at room temperature in all measurements. Contact angle measurements were conducted using both the free software ImageJ (https://imagej.net) and the open-source software tool “Dropen”.^38^ The surface morphology of the laser-ablated hydrophilic tracks was examined using a field emission scanning electron microscope (SEM, Zeiss, Germany) at an accelerating voltage of 5.00 kV. Before observation, the samples were sputtered with thin gold layers to enhance their contrast since the hydrophobic coating is not electrically conductive.

#### Natural and Artificial Weathering Tests

Several artificial and natural weathering tests were performed in both lab-sized waterjet and corrosion chambers as well as in Miami, Florida (U.S.). The test samples (token) have a diameter of 45 mm, and the wetting properties for all samples have been recorded as well as the ability of the contrast wettability surface sample to transport water along the edge of the token.

The natural weathering test took place in Florida (USA) due to its unique combination of high sunlight irradiation together with high year-round temperatures, as well as high rainfall and humidity. A simultaneous combination of these conditions creates a harsh climate for materials, which establishes the area as an international benchmark for the automotive industry. In this study, 12 circular test tokens were exposed to harsh natural weathering conditions in Florida because there has been observed a faster production of degradation than in more northern locations. The samples followed the ASTM G7 outdoor test protocol. For this test, a direct exposure mounting method has been used. Figure 4a shows the direct mounting method, the flat rigid panel (plywood is used), and it is fixed in a self-supporting aluminum frame in a way that their front surface faces the sun and has no cover, which means that samples are affected by all elements of the atmosphere. Specimens are fixed by aluminum clamps which cover 1 cm of the top and bottom side of each sample (token). Any sample exposed outdoors will receive more solar energy when the sunlight beam hits the sample directly than when it comes from an angle. In this study, the exposure angle (known as latitude angle) is varied aiming to maximize the sunlight dosage, with the following schedule: September and March from 25° to 34°, April to August will be 5°, October to February will be 45°. The minimum tilt angle of 5° is selected to allow the water to run off and not accumulate on the sample (token) surface. This angle also collects more dew and moisture retains longer than the 45°. Each month a test token was collected from the field for wettability and water transport analysis.

To evaluate the durability of different materials and/or microstructures to corrosion, the samples have been exposed to a natural spray test in accordance with ISO 9227 2012 (Natural Spray Test Standard), ISO 8407:2009 (corrosion of metal and alloys), and ASTM B117-11 Standard Practice for Operating Salt Spray Apparatus. A corrosion chamber, model CCT EC014 (Q-Lab, Bolton, United Kingdom), has been used for designing and development testing (more details in Supporting Information). For both samples, the wettability is measured before the test. On test completion (2000 h of corrosion testing), the samples have been washed with deionized water and the wettability of the samples is measured again, as well as the maximum volume of water that the contrast wettability surface sample (token) can transfer along the hydrophilic tapered tracks without accumulating in the centers of the token.

To extend the validation tests, an in-house waterjet has been used for both samples. The nozzle diameter of the waterjet was 3 mm, the exit speed of the water was approximately 12 m/s, the Weber number was ∼6100, and the Reynolds number was ∼36 200. Both samples were exposed for up to 264 h.

## Supporting Information Available

Estimation of water absorption capacity; SEM image and EDX analysis of contrast wettable surface; variation of the apparent contact angles on the hydrophilic tracks after exposing to vapor for different length of time; surface curvature measurements; artificial and natural weathering test conditions and their effects on wetting properties of the hydrophobic coating; schematic of water jet test setup and water contact angle measurements of the hydrophobic coating against exposure of water jet at different lengths of time; photographs of the corrosion chamber and test conditions inside the chamber; parameter of femtosecond laser used in this study; ablation depth analysis of the microscale features using laser confocal microscope; table summarizing the list of durability tests conducted on contrast wettable surface and their outcomes (pass/fail) (PDF)

## Notes

T.D. and T.M. work for FT Technologies UK Ltd. while collaborating with universities on SURFICE Marie Skłodowska-Curie project. T.D. and T.M. have no employment, financial, or personal conflict of interest with other organizations.
