# Supplementary material for: Capillary-Driven Water Transport by Contrast Wettability-Based Durable Surfaces
Source: ACS Appl Mater Interfaces. 2023 May 26;15(22):27206–13. doi: 10.1021/acsami.3c03840 (PMC10251349; doi:10.1021/acsami.3c03840)
Supplement: Supplementary file 1 — am3c03840_si_001.pdf [file am3c03840_si_001.pdf]

Supporting Information

Capillary-driven water transport by contrast wettability-based durable surfaces

Theodoros Dimitriadis[1,3†], *Luca Stendardo*[2†], *Irene Tagliaro*[2], *Anna M. Coclite*[1], *Carlo Antonini*[2*],

Tanmoy Maitra[3*]

[1]Institute of Solid-State Physics, Graz University of Technology, Graz, 8010, Austria.

[2]Department of Materials Science, University of Milano-Bicocca, via R. Cozzi 55, 20125. Milano, Italy.

[3]Department of Engineering, FT Technologies (UK) Ltd., Sunbury-on-Thames, TW16 7DX, United Kingdom.

*corresponding author's email addresses: tanmoy.maitra@fttechnologies.com, carlo.antonini@unimib.it

Supporting Information

S1. Single Droplet Absorption by Hydrophilic Track

To further characterize the phobic-to-philic transition on the contrast wettability surface, droplets have been deployed close to the hydrophobic-hydrophilic junction. The goal of this analysis was to investigate up to which volume droplets could be absorbed at once by the hydrophilic track, without leaving water residues on the hydrophobic surface.

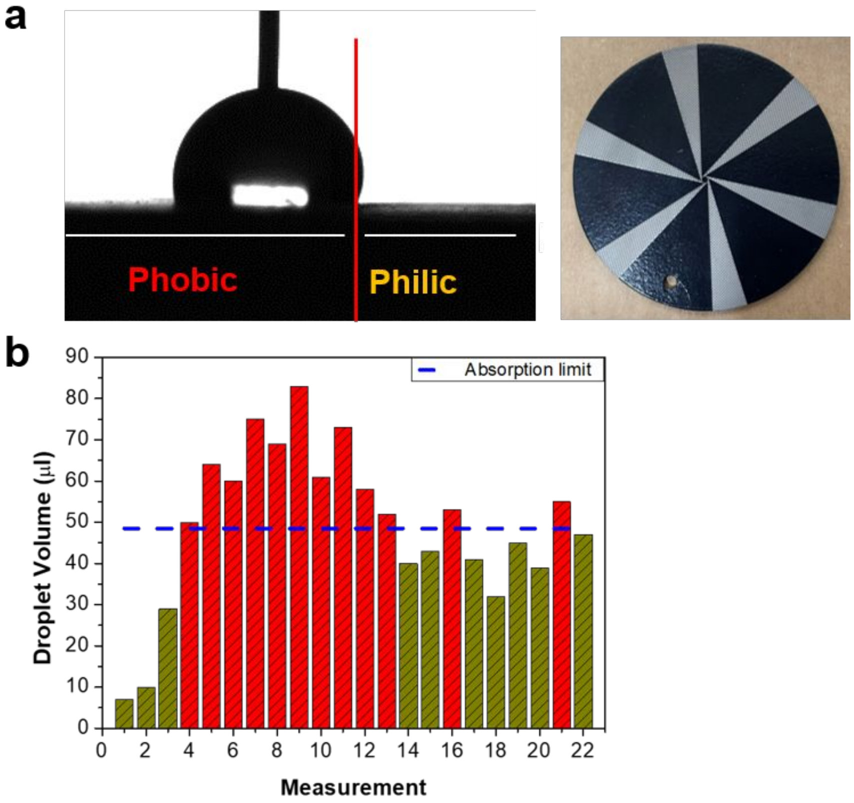

Figure S1: Characterization of phobic-to-philic transition. a) Deployment of a droplet on the hydrophobic surface, close to the hydrophobic/hydrophilic junction. The droplet volume was gradually increased using a syringe pump and the droplet volume at the moment of absorption was recorded. b) 22 measurements were performed in total. Whenever the droplet was absorbed completely the bar is colored green, while a red bar represents the presence of water residues on the hydrophobic surface after absorption. The blue line represents the estimated volume limit for full absorption.

Figure S1a shows how the water was deployed close to the junction: a syringe pump was used to deploy water at a constant volume rate on the hydrophobic surface. At a given instant, the droplet would get in touch with the hydrophilic surface due to its growth in volume and it would be absorbed by it. At that point, the volume was recorded from the syringe pump and the hydrophobic section was inspected for residual water. If the hydrophilic track did absorb the droplet completely, then a green bar is represented in Figure S1b, otherwise, it is represented in red. After a total of 22 measurements, the absorption limit was estimated to be close to the 50 µL mark.

S2. The geometry of the Hydrophilic Tracks

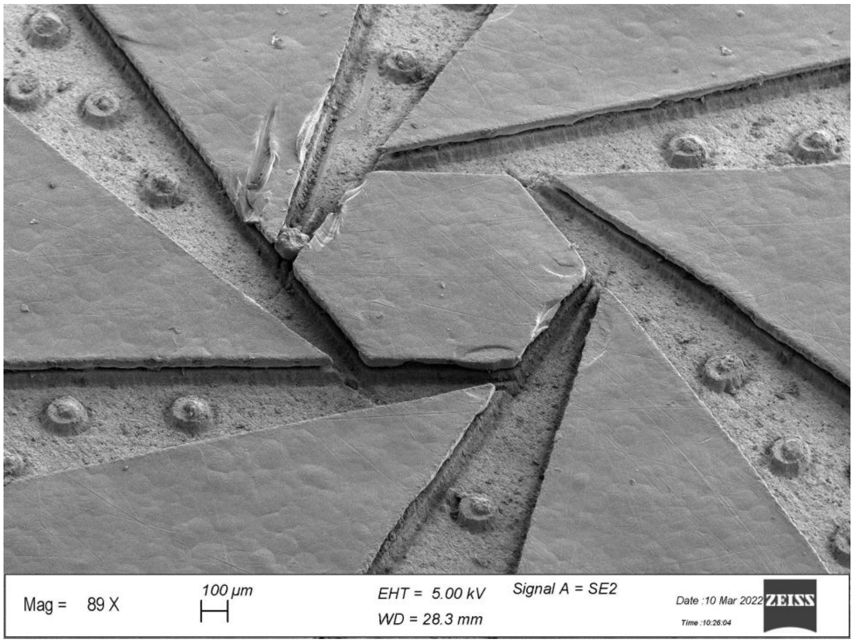

Figure S2: The centre of the contrast wettability surface. SEM image of the axes of symmetry, that are diverging by 1.8° and form a polygon in the centre of the token.

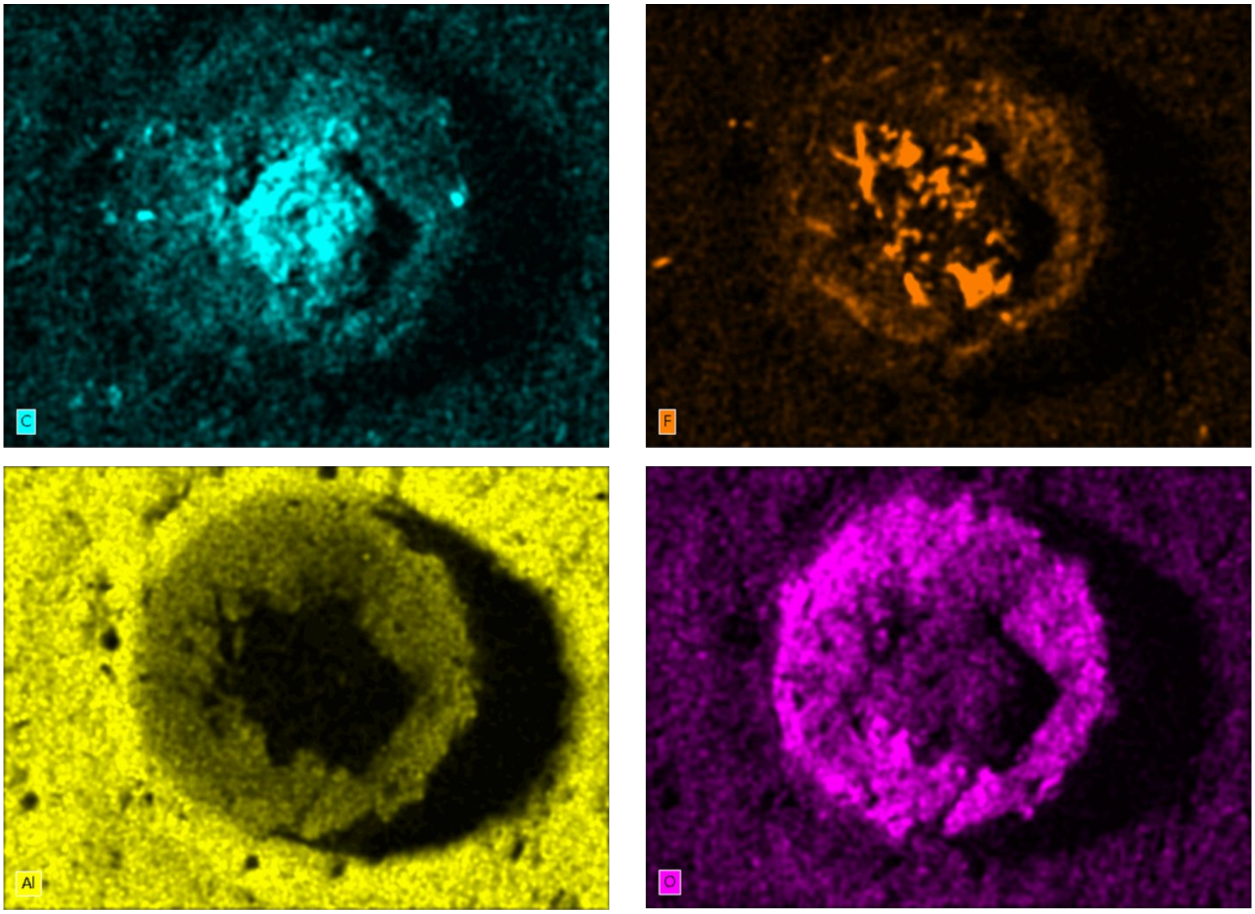

Figure S3: EDS analysis of the micropillar. The four images display each element captured from the EDS analysis.

S3. Variation of the Apparent Contact Angle on the Hydrophilic Tracks in Different Conditions

It was observed that the water contact angle on the hydrophilic tracks is dependent on the water content that is inside the micro-structure of the tracks. To investigate how the contact angle changes depending on the quantity of water inside the micro-structure, the sample (test token) was exposed to vapor before taking the contact angle measurements. It was done by heating water inside a 250 mL baker to 80 °C and exposing the sample (token) to its vapors for a different amount of time (0, 2.5, 5, 10, 15, and 20 seconds).

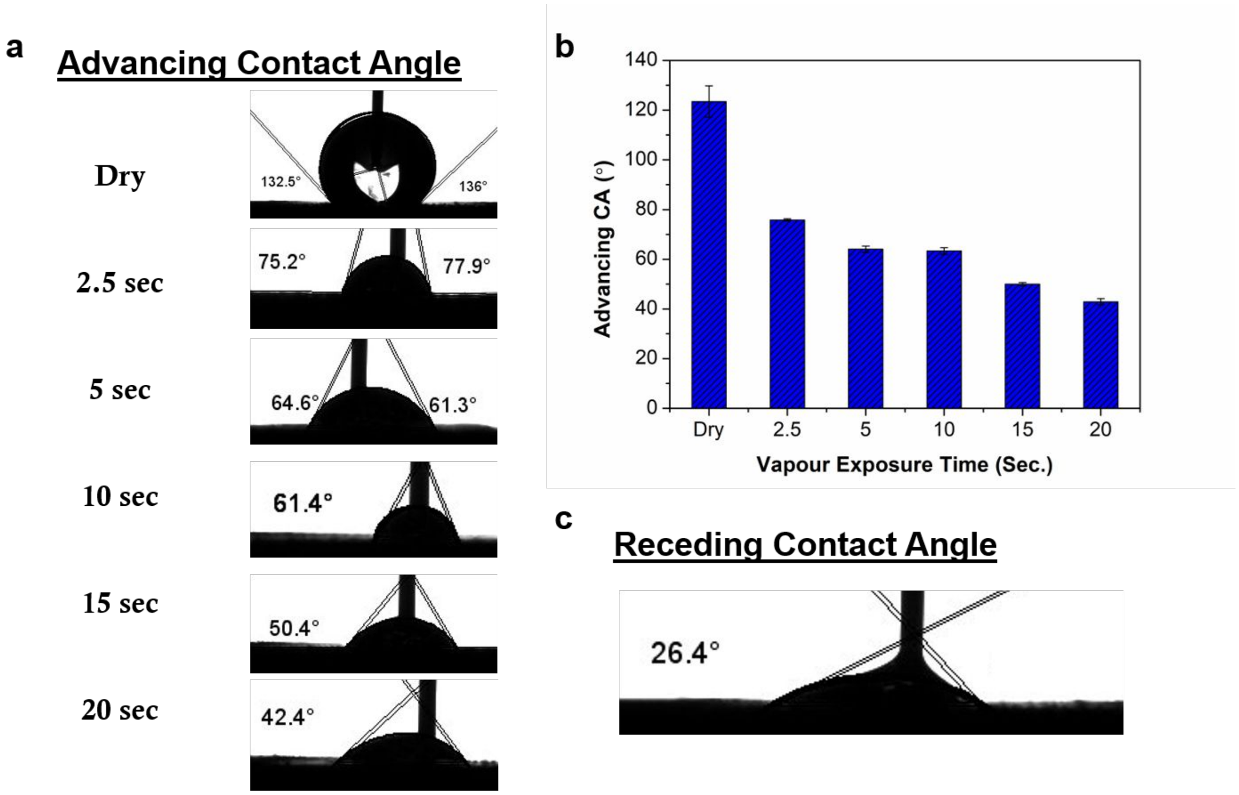

Figure S4: Water contact angle dependence of the water content in the hydrophilic track. a) Evolution of the droplet shape on the hydrophilic track for different vapor exposure times. It can be seen that to classify as "hydrophilic", the track needs to be exposed to a minimum amount of vapor, which in this work was found to be 2.5 seconds. b) Summary of the advancing contact angles measured on the hydrophilic tracks depending on the vapor exposure time. c) Measurement of the receding contact angle, which was found to be constant in all cases.

Figure S4a shows the advancing contact angle and the shape of the droplet on a hydrophilic track as a function of the vapor exposure time. It can be noted that on a completely dry surface the advancing contact angle is > 90°, while for every other case, it is < 90°. The graph in Figure S4b summarizes the advancing contact angle values for different vapor exposure times. Once completely wetted, the tracks show a strong hydrophilic character, which can be seen from the measurements of the receding contact angle, which was found to be constant at 26° for all the vapor exposure times (Figure S4c).

S4. Surface Curvature Measurements

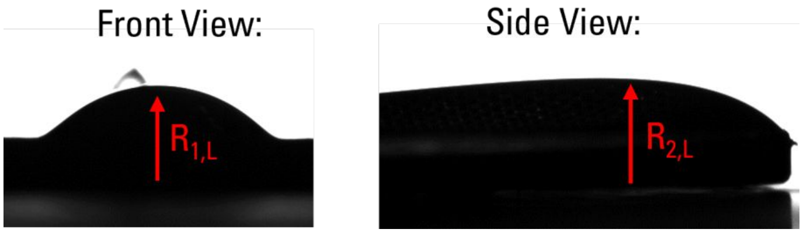

Figure S5: The shape of the water accumulation on the hydrophilic tracks. Water was not removed from the contrast wettability surface, since the test was performed on the flat test sample (token). The radii of curvature $R_{1,L}$ and $R_{2,L}$ were used to calculate the Laplace pressure inside the water accumulation.

By recording the radii of curvatures $R_{1,L}$ and $R_{2,L}$ as a function of the accumulated volume, it was possible to derive the graph presented in figure 3b of the main text, plotting P_T vs. Collected water. The radii of curvature were found by using the “Kappa - Curvature Analysis” plugin that comes as part of the “Fiji” application, available for free on https://imagej.net/software/fiji/downloads.

S5. Artificial and natural weathering tests

The samples followed the ASTM G7 outdoor test protocol. For this test, a direct exposure mounting method has been used. Each month a contrast wettability surface sample was collected and measured.

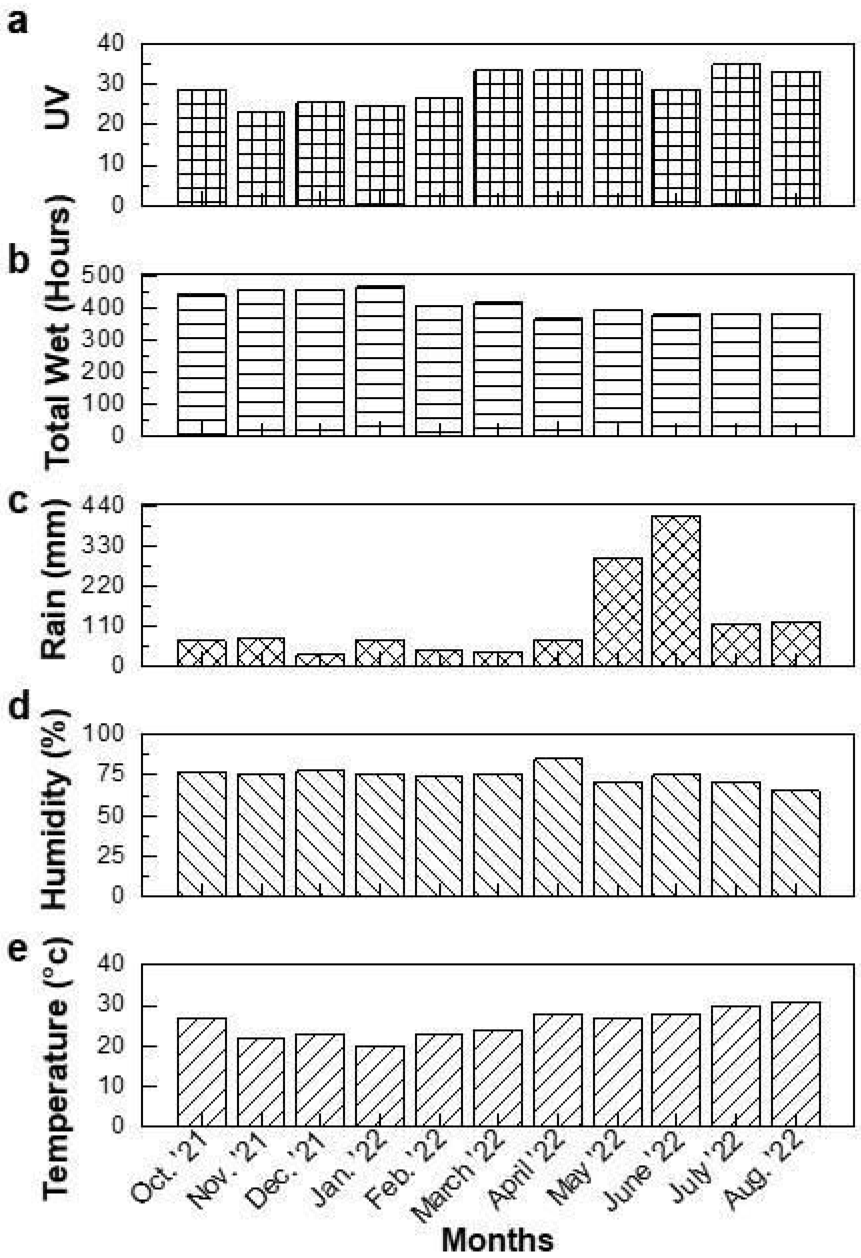

Figure S6: Florida's average meteorological data. The figure shows Florida's weathering conditions such as: (a) UV, (b) total wetness, (c) rain, (d) humidity, and (e) temperature from the meteorological data collected for each day for all months. The average value of all days for each month is represented accordingly.

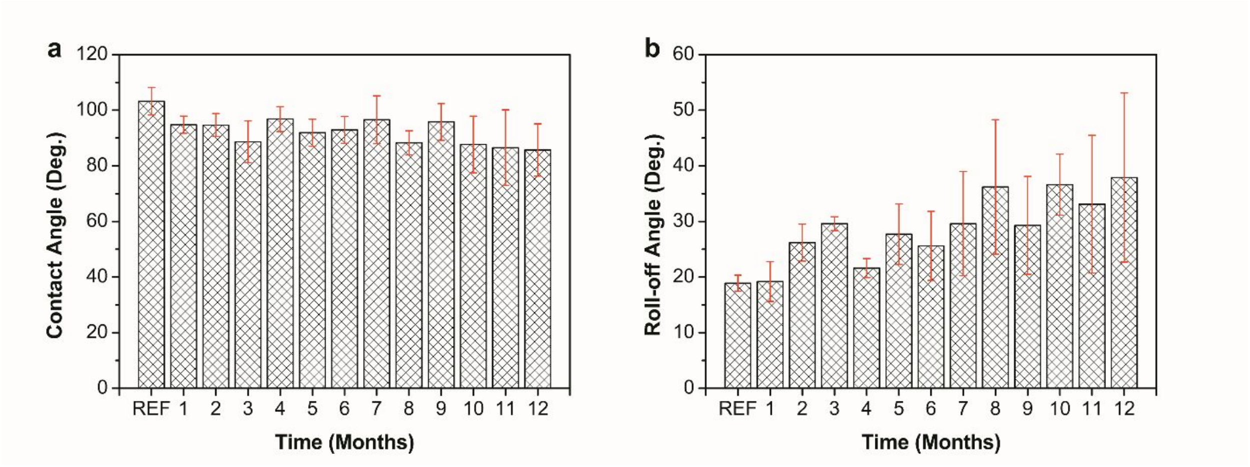

Figure S7: Influence of Florida's annual weathering conditions on wettability. After a long exposure of the Contrast wettability samples in Florida's weather, it is feasible to compare the long-term influence of harsh natural weather conditions on the contrast wettability surface, especially on the polymers (hydrophobic) surface. The graphs show that the water contact angle (a) and roll-off angle (b) are not significantly affected.

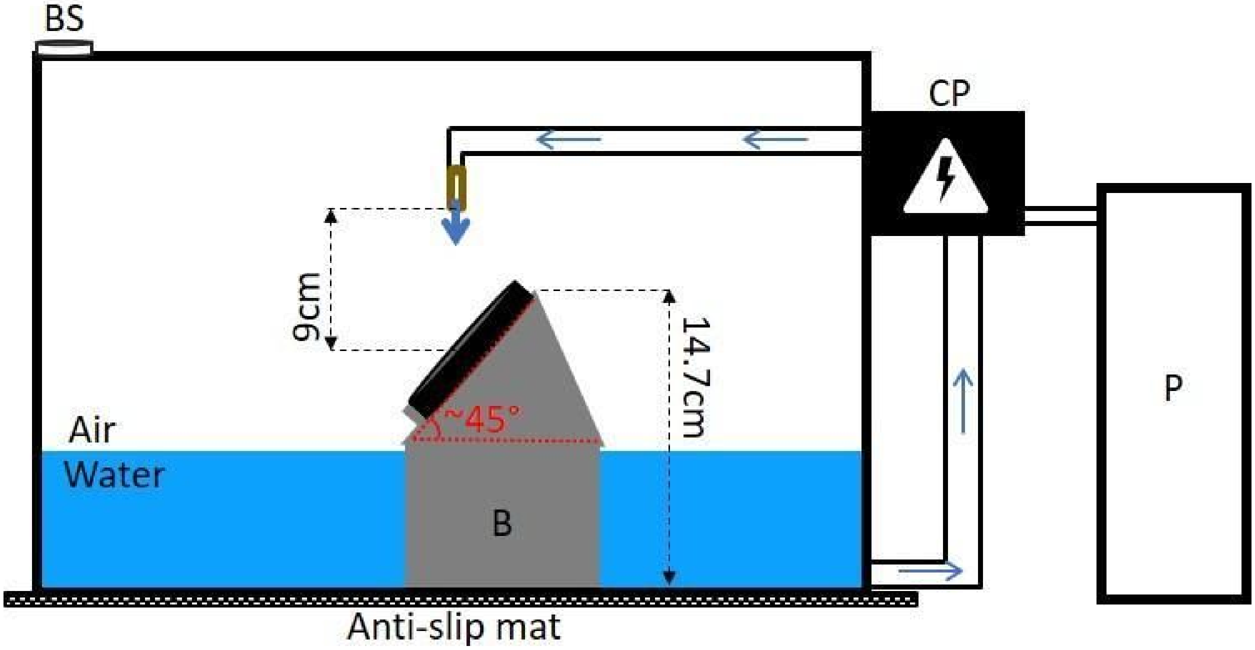

Figure S8: Schematic illustration of the waterjet. The whole system is located inside another container to prevent leak issues. The system lies on top of an anti-slip mat to further contaminate any small leakage.

Starting from right to left; P is the pressure system (0.4 bar), CP is the centrifugal pump, the diameter of the nozzle is 3mm and B is the holder of the samples in this case of the sample (tokens). On the top left corner, BS is the bubble spirit. Due to the inclined base, the waterjet droplets hit the sample surface with a maximum droplet force of 3.44 N.

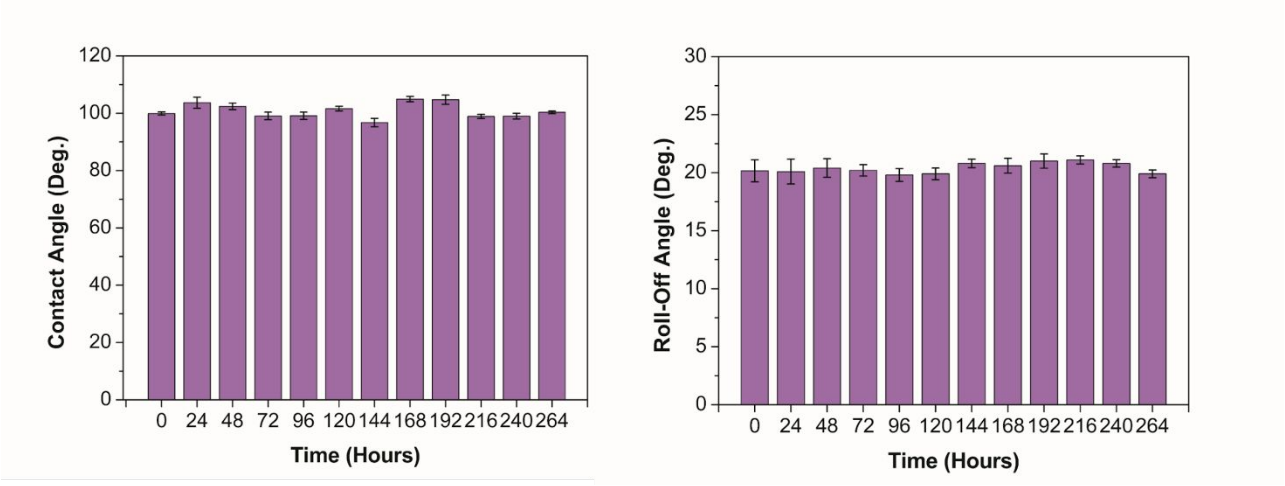

Figure S9: Durability of Contrast Wettability Token under Waterjet. Contrast wettability samples prove the durable contrast wettability surface since the water contact angle (a) and roll-off angle (b) do not have been affected significantly.

In order to test the samples in the corrosion chamber, deionized water has been used from an external tank for the preparation of the solution. It is ensured that the water conductivity is less than or equal to 20 µS/cm at 25ºC ±2 ºC, whereas the concentration of sodium Chloride is 50g/L ±5g/L. The preparation method is the following: a clean graduate bucket is used in the testing room, the solution has to be mixed,

this process shall last for a couple of minutes in order to dissolve the salt completely and a test of pH level has been performed on the solution. The values shall be between 6.5 and 7.2 at 25ºC and recorded. Next, the contrast wettability surfaces shall be placed inside the chamber and fixed accordingly to their mounts as shown in Figure S10. The temperature in the corrosion chamber during the experiment is 35ºC ±2 ºC, the concentration of sodium Chloride is 50g/L ±5g/L and the air pressure is 0.7- 1.0 bar.

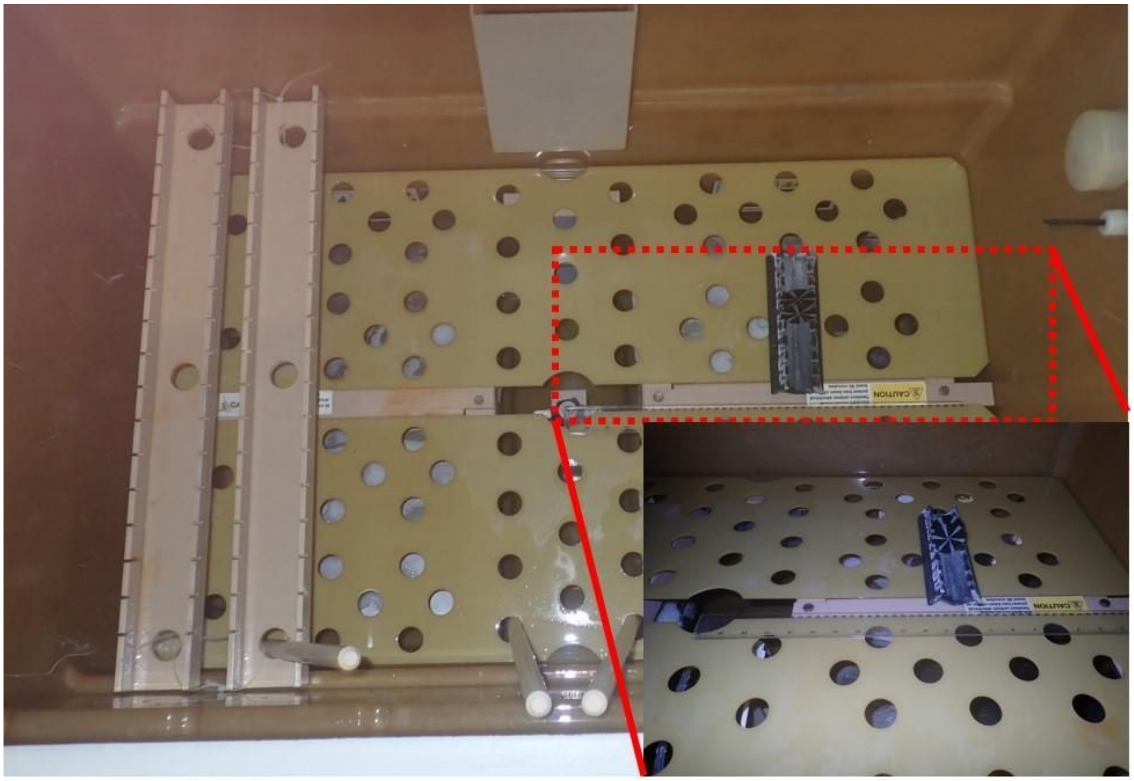

Figure S10: Image from the inner part of the Corrosion Chamber focused on the samples. The contrast wettability surface samples are placed 30 cm away from the nozzle of the chamber. Both samples will remain inside the chamber and will be exposed for up to 2000 hours.

S6. Parameters of the Femtosecond laser

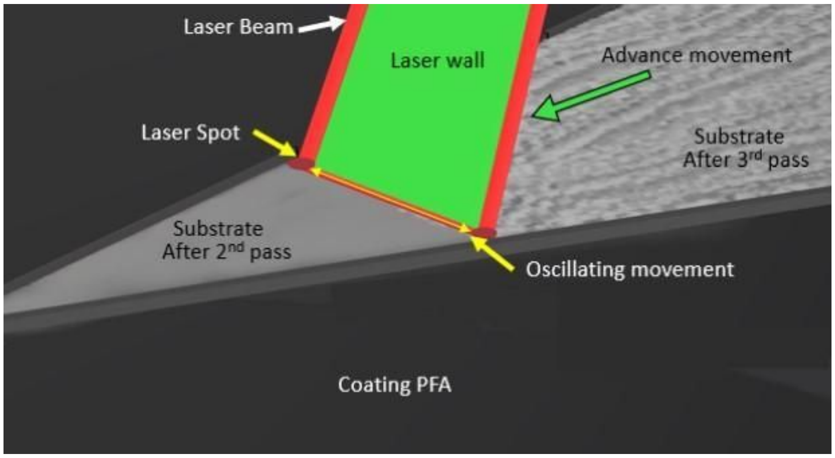

Figure S11: Illustration of the laser ablation while the laser beam passes 3 times over the selected area.

After the third pass, the PFA coating has been removed and the exposed hard-anodized Al substrate is ready for the micro-texturing of the pillars. It is shown that due to the oscillating movement of the laser beam, it is possisble to find PFA residuals on top of the exposed hard-anodized Al substrate, which has not been removed successfully by the vacuum system.

Parameters	Values

Wavelength (λ)	1035 nm
Power (P)	40 W
Minimum Pulse Energy	80 µ
Repetition rate	50 MHz

Table S1. Process parameters of the SW femtosecond laser.

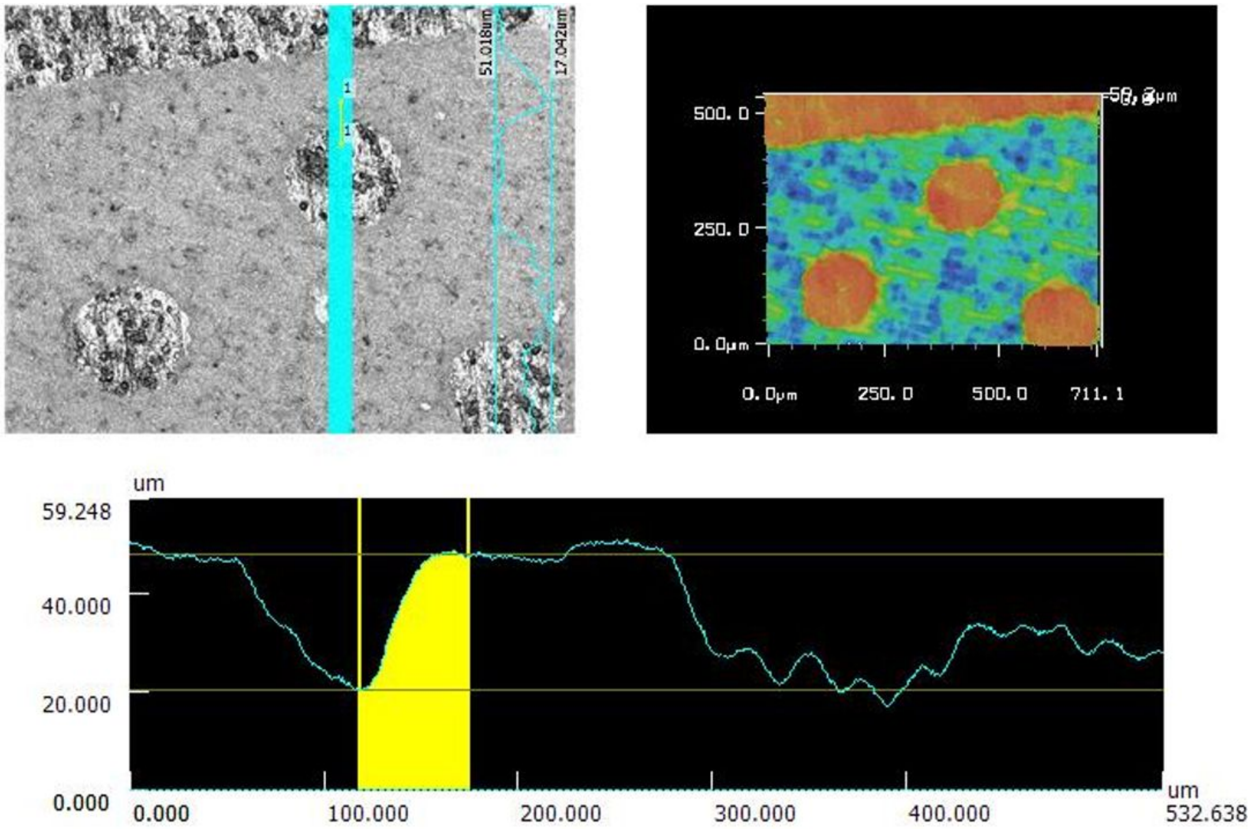

Profile1	Horz. dist.	Hght. diff.	Hght. ave.	Angle	C.S. length	C.S. area	R	Comment
All	532.638um	22.323um	35.502um	2.400°	613.774um	18934.278u...		
Seg.1	55.274um	27.221um	38.489um	26.219°	67.854um	2164.990um2		
Seg.2								
Seg.3								

Figure S10: Pillars height profile. The image shows the pillar height profiles and measurements contacted by the laser.

Test	Conditions	Test time	Pass/fail status	Water Transportation Status
Corrosion Test	Neutral Salt Spray (ASTM B117)	2000 hours	Pass	Successful
Outdoor Weather Exposure Test (Benchmark location Florida U.S.)	UV (>30 MJ/m^2)	12 Months	Pass	Successful
	Total Wet (>350 Hours)			
	Rain (>50mm)			
	Humidity (>55%)			
	Temperature (>20 °C)			
Waterjet Test	impinging velocity<br>V_0= 12 m/s, We~ 6100	264 hours	Pass	Successful

Table S2. The table shows the advantage of the contrast wettability surface. The contrast wettability surface successfully passed all the tests (indoor and outdoor) and showed no degradation regarding the transportation of water.
